# Social determinants of common metabolic risk factors (high blood pressure, high blood sugar, high body mass index and high waist-hip ratio) of major non-communicable diseases in South Asia region: a systematic review protocol

**DOI:** 10.1186/s13643-017-0576-6

**Published:** 2017-09-07

**Authors:** Sudesh Raj Sharma, Shiva Raj Mishra, Kusum Wagle, Rachel Page, Anna Matheson, Danielle Lambrick, James Faulkner, David Lounsbury, Abhinav Vaidya

**Affiliations:** 1 Nepal Development Society, Kathmandu, Nepal; 2 James P. Grant School of Public Health, BRAC University, Dhaka, Bangladesh; 3 College of Health, Massey University, Wellington, New Zealand; 4 University of Southampton, Southampton, UK; 5 University of Winchester, Winchester, UK; 6 Albert Einstein College of Medicine, Bronx, NY 10461 USA; 7 Kathmandu Medical College, Kathmandu, Nepal

**Keywords:** NCDs, Risk factors, Social determinants, South Asia

## Abstract

**Background:**

Prevalence of non-communicable diseases has been increasing at a greater pace in developing countries and, in particular, the South Asia region. Various behavioral, social and environmental factors present in this region perpetuate common metabolic risk factors of non-communicable diseases. This study will identify social determinants of common metabolic risk factors of major non-communicable diseases in the context of the South Asian region and map their causal pathway.

**Methods:**

A systematic review of selected articles will be carried out following Cochrane guidelines. Review will be guided by Social Determinants of Health Framework developed by the World Health Organization to extract social determinants of metabolic risk factors of non-communicable diseases from studies. A distinct search strategy will be applied using key words to screen relevant studies from online databases. Primary and grey literature published from the year 2000 to 2016 and studies with discussion on proximal and distal determinants of non-communicable risk factors among adults of the South Asia region will be selected. They will be further checked for quality, and a matrix illustrating contents of selected articles will be developed. Thematic content analysis will be done to trace social determinants and their interaction with metabolic risk factors. Findings will be illustrated in causal loop diagrams with social determinants of risk factors along with their interaction (feedback mechanism).

**Discussion:**

The review will describe the interplay of social determinants of common NCD metabolic risk factors in the form of causal loop diagram. Findings will be structured in two parts: the first part will explain the linkage between proximal determinants with the metabolic risk factors and the second part will describe the linkage among the risk factors, proximal determinants and distal determinants. Evidences across different regions will be discussed to compare and validate and/or contrast the findings. Possible bias and limitations of this study will also be discussed.

**Systematic review registration:**

PROSPERO CRD42017067212

**Electronic supplementary material:**

The online version of this article (10.1186/s13643-017-0576-6) contains supplementary material, which is available to authorized users.

## Background

**Fig. 1:**
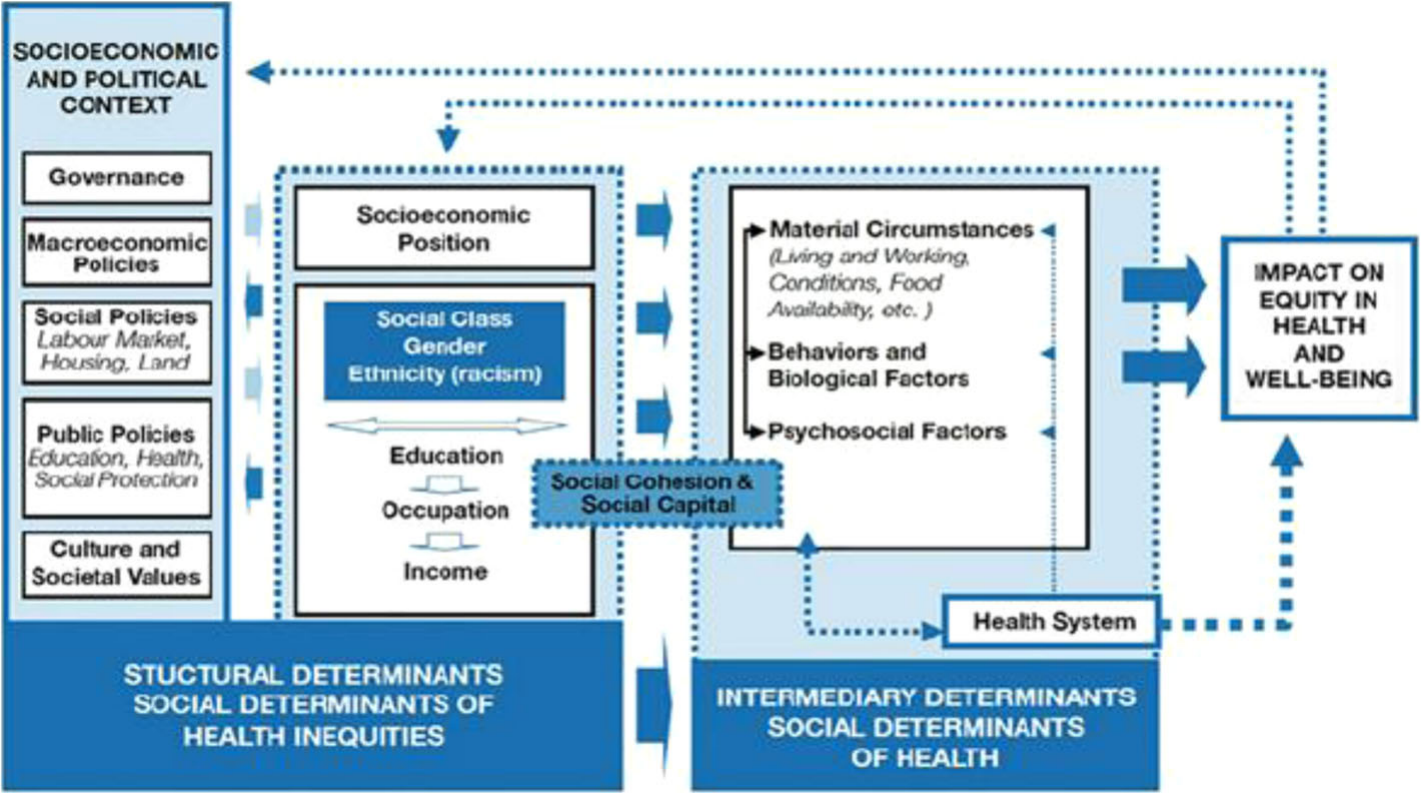
Social determinants of health framework proposed by the WHO Commission on SDH (link, http://www.who.int/sdhconference/resources/ConceptualframeworkforactiononSDH_eng.pdf)

Global burden of non-communicable diseases (NCDs) has been increasing, and the South Asia region in particular is showing a rapid increase [1]. In this region, nearly half of the disease burden and two-thirds of the total deaths are caused by NCDs [2]. In addition to mortality, disability due to NCDs is posing a serious economic burden due to reduced productivity and poverty among affected people who need long-term care [3, 4]. Cardiovascular diseases, respiratory diseases, cancers and diabetes are four major diseases causing deaths and creating burden in this region [1]. Common risk factors for them are poor dietary habits, tobacco use and inadequate physical activity and metabolic risk factors such as high blood pressure, high blood sugar, high Body mass index (BMI) and high waist-hip ratio (WHR) [5–8] [Additional file 1: Appendix 1]. NCDs have a complex causal mechanism characterized by having multiple etiologies and factors [9]. Factors causing NCDs are influenced by an individual’s behavior along with its interaction with the external socio-economic environment, on the top of the genealogical predisposition that people are born with [10, 11]. Thus, changing individuals’ behavior is not adequate in itself without addressing underlying the social, economic, environmental and cultural factors collectively known as social determinants of health (SDH) [12]. A SDH framework has been proposed by the World Health Organization to elucidate these complexities [13]. This framework is based on *Social Production of Disease Theory* and integrates the best available evidences on social determinants of health. The framework is increasingly being utilized to understand and address multifactorial and complex causation of health problems like NCDs [12, 14, 15]. In the framework, the proximal determinants include behavioral and psychosocial factors, material circumstances, community capital and cohesion and health system factors whereas distal determinants include social positioning/class and socio-economic context (Fig. 1).

The lack of progress in addressing these social determinants of complex health problems indicates that our existing approaches are inadequate and may need help from an alternative science. Complexity sciences or system sciences hold the key to addressing these complex public health problems by improving our existing public health approaches [16, 17]. System sciences methods and tools are well placed for understanding the dynamic structure and emergent behavior of a social system leading to complex health problems. Frameworks like SDH could be further improved and contextualized through identifying gaps in practice, and enable better design of effective interventions if a complex systems approach is applied [18].

There is a need to understand social determinants of common metabolic risk factors of major NCDs for systematic action in the South Asia region using system science approach and tool. However, there is a scarcity of such comprehensive systematic reviews on social determinants of common metabolic risk factors in the South Asia region. This systematic review will first identify the common social determinants of major NCDs and then map the causal pathways through which those determinants interact to perpetuate common metabolic risk factors (high blood pressure, high blood sugar, high BMI and high WHR) among the adult population in South Asia.

## Methods

The systematic review will be conducted following the Cochrane guidelines [19]. A Preferred Reporting Items for Systematic Reviews and Meta-Analyses for Protocols (PRISMA-P) checklist has been completed to ensure that the protocol is robust [20] [Additional file 2: Appendix II]. We will use thematic content analysis to identify social determinants of NCD risk factors and map the causal pathways leading to major NCDs in the South Asia region using the WHO framework described earlier.

### Search strategy

We will search for relevant published articles and grey literature through PubMed, Embase, Web of Science and ProQuest Central using sequence of key words listed in Table 1. A detailed search strategy using Boolean operators and contractions for key search terms will be developed in consultation with a librarian at the Massey University library in Wellington.

**Table 1:** Key words for searching articles for the review

Key words	Other variations of key words	
NCDs metabolic risk factors plus proximal determinants plus distal determinants plus geographic location	NCDs metabolic risk factors	High blood pressure, hypertension, high blood glucose, hyperglycemia, diabetes, high BMI, obesity, overweight, waist circumference, waist-hip ratio
	Proximal determinants	Determinants, risk, diet, salt intake, tobacco, smoking, alcohol, physical inactivity, lifestyle
	Distal determinants	Social determinants, education, income, gender, race, caste, ethnicity, socio-economic status, wealth index, income
	South Asia	Nepal, India, Bangladesh, Pakistan, Sri Lanka, Bhutan, Maldives, Afghanistan

### Inclusion criteria

Inclusion criteria for selection of studies and grey literature will be as follows: (a) primary research and grey literature published between 2000 and 2016, (b) studies among an adult population aged 20–65 from South Asian region (Nepal, India, Bangladesh, Pakistan, Sri Lanka, Bhutan, Maldives, Afghanistan) and (c) studies and literature that have generated empirical evidences on proximal and/or distal determinants of the selected NCD risk factors.

Both published and unpublished, grey and peer reviewed, and qualitative and quantitative studies will be included. Any study outside of the South Asia region and not mentioning at least one of the proximal and/or one of the distal determinants from the SDH framework will be excluded [List in Additional file 3: Appendix III].

### Screening procedures

Studies will be primarily screened on the basis of inclusion criteria. Two independent reviewers (SRS and KW) will review the title and abstract checking all the inclusion criteria. We will use Covidence (https://www.covidence.org/), a Cochrane Technology Platform tool to systematize the process. The secondary screening will involve full-text review, review of methods including selected cutoff values for metabolic risk factors [Additional file 1: Appendix I] and review of findings and discussion section for causal pathway analysis on social determinants of NCD risk factors. The team will exclude the duplicated articles and then finalize the list for systematic review.

### Quality assessment of research papers

The selected articles will be assessed for their quality using QUALSYST tool [21]. There are many tools (for example, Effective Public Health Practice Project-EPHPP and National Institute of Health Quality Assessment Tool for quantitative studies; Popay et al. criteria and Mays et al. criteria for qualitative studies) to assess the quality of research papers [22–25], but limited tools/criteria have incorporated checklists for both qualitative and quantitative research designs. QUALYSYST tool has been validated for quality assessment of both qualitative and quantitative studies for systematic review. Further, the tool is easy to use and draws upon existing tools to address the issue of simultaneous assessment of the quality of studies which are diverse in nature. Two independent researchers (SRS and KW) will assess the quality of the studies and exclude papers with low quality score (Inter-rater agreement score of < 0.55). The final list will be agreed upon by both researchers (SRS and KW) for data extraction and analysis.

### Data extraction and management

Data will be extracted from the final list of articles by the research team in an illustrative matrix as shown in Table 2.

**Table 2:** Example of illustrative matrix of selected articles

Literature type and year of publication	Research type	Study objective/method	Study location and sample size	Metabolic risk factor	Proximal determinants	Distal determinants	Causal linkage thematic analysis
Primary article or grey literature as searched in three databases/year of publication as mentioned	Quantitative or qualitative or mixed-method research	Describe in detail about the study objective and methods	Country and region where the study was taken and sample size	Metabolic risk factor under study	See Additional file 3: Appendix III	See Additional file 3: Appendix III	Coding and thematic analysis about the possible causal linkage among determinants based on the results and discussion of the article

### Data analysis including mapping of causal pathways

Thematic analysis will be performed to identify patterns/themes across the extracted data/codes and stream out common pathways from determinants to risk factors of NCD simultaneously to data extraction. The team will meet regularly to check the consistency in data extraction and analysis and discuss issues, if any.

The identified causal linkages/pathways will be further consolidated as per the SDH framework and mapped in the form of causal loop diagram (CLD). CLD is a system dynamic mapping tool to express the dynamic causal relationships operating among variables giving rise to a specific situation. It is based on control and feedback theory and mainly driven by the endogenous behavior of the variables in generating a specific problem and how the problem, in return, is providing feedback to the determinants. The CLD from the review will show the linkages between social determinants of common NCD risk factors, and how these determinants reinforce or balance each other. The strategy to construct CLD in this review will be based on *Business Dynamics*, a system science resource [26].

**Fig. 2:**
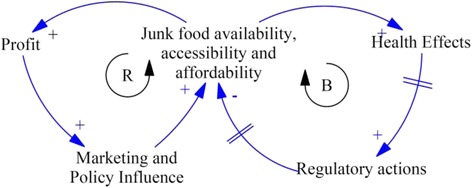
Example of causal loop diagram of an issue of *Junk Food control*

An example of casual loop diagram depicting the issue of *Junk Food control* is presented below. There are two loops in the diagram, one is balancing loop indicated by *B* in the centre of the loop and the other is the reinforcing loop indicated by *R* in the centre, see Fig. 2.

The balancing loop is a goal-seeking loop which tries to bring down the availability and affordability of the junk food however is unable to do so due to system delays (indicated by // sign in the arrow). The positive linkage (+ sign in the arrowhead) between *Junk Food availability, accessiblity and affordability* and *Health effects* indicates that the universal availability and affordability of junk food is giving rise to health effects. The increasing health effects are in-turn providing pressure to the authorities to develop regulatory policies which are delayed. These regulatory policies again have delayed implementation and are often in action when the problem has already grown out of proportion with other unintended consequences. On the left-hand side of the figure, the reinforcing loop indicates how the junk food companies utilize their profits to bolster marketing of their products and influence policy for delayed implementation of regulatory policies inorder to maintain their sales and profit. The causal loop diagram will be built using the “Vensim”, a freely available software program for developing system models [27].

### Discussion

The findings will describe how various social determinants interact and influence each other in the South Asian population brewing and propagating an epidemic of common metabolic risk factors of NCD. Our review will depict such interactions within and outside of the health and social systems using a causal loop diagram based on qualitative analysis of selected articles. The review will be structured in two parts. The first part would explain the linkage between proximal determinants with the metabolic risk factors. The second part will describe the linkage among distal determinants, proximal determinants and the metabolic risk factors of major NCDs in this region. Regional and country wise comparison will be done to validate and/or contrast the findings. Causal loop diagrams of social determinants of common metabolic risk factors of NCDs will provide insights on how the social determinants are linked with each other in perpetuating NCD problem for the South Asia region context. The review will have a much needed policy and programmatic implications in the current situation of increasing burden of NCD in South Asia. However, this review will be limited to articles written in English language and identified through four online databases.

## Additional files

Additional file 1: Appendix I: Cutoff values of metabolic risk factors of NCDs (DOCX 15 kb)

Additional file 2: Appendix II: PRISMA-P checklist for the systematic review (DOCX 21 kb)

Additional file 3: Appendix III: List of proximal and distal determinants (based on SDH framework) (DOCX 16 kb)

## Abbreviations

BMI: Body mass index
CLD: Causal loop diagram
NCDs: Non-communicable diseases
PRISMA-P: Preferred Reporting Items for Systematic Reviews and Meta-Analyses for Protocols
SDH: Social determinants of health
WHO: World Health Organization
WHR: Waist-hip ratio
