# Supplementary material for: Social determinants of common metabolic risk factors (high blood pressure, high blood sugar, high body mass index and high waist-hip ratio) of major non-communicable diseases in South Asia region: a systematic review protocol
Source: Syst Rev. 2017 Sep 7;6:183. doi: 10.1186/s13643-017-0576-6 (PMC5590129; doi:10.1186/s13643-017-0576-6)
Supplement: Supplementary file 1 — Appendix I: Cutoff values of metabolic risk factors of NCDs (DOCX 15 kb) [file 13643_2017_576_MOESM1_ESM.docx]

**Additional file 1: Appendix I: Cut-off values of metabolic risk factors of NCDs**

| **Metabolic risk factors** | Cut-off values | | | | |
| --- | --- | --- | --- | --- | --- |
|  | **WHO (1999)[1]** | **EGIR (1999)[2]** | **NCEP ATP III[3]** | **AACE (2002)[4]** | **IDF (2006)[5]** |
| High Blood Pressure | >=90 mm Hg (Diastolic BP) or >=140 mm Hg (Systolic BP) | >=90 mm Hg (Diastolic BP) or >=140 mm Hg (Systolic BP) | >=90 mm Hg (Diastolic BP) or >=140 mm Hg (Systolic BP) | >=85 mm Hg (Diastolic BP) or >=130 mm Hg (Systolic BP) | >=85 mm Hg (Diastolic BP) or >=130 mm Hg (Systolic BP) |
| High Blood Sugar (Fasting Plasma Glucose level) | >=6.1 mmol/l (110mg/dl) | >=6.1 mmol/l(110mg/dl) | >=6.1 mmol/l (110mg/dl) | >=6.1-6.9 mmol/l (110-125mg/dl) | >=5.6 mmol/l (100mg/dl) |
| High BMI | >=30 kg/m^2^ value | >=30 kg/m^2^ value | >=30 kg/m^2^ value | - | >=30 kg/m^2^ value |
| High Waist-Hip Ratio | >=90 mm for Men and >=85 for Women | >=94 mm for Men and >=80 for Women | >=102 mm for Men and >=88 for Women | - | >=90 mm for Men and >=80 for Women  (Asian origin) |

**References:**

1. Alberti KGMM, Zimmet Pf: **Definition, diagnosis and classification of diabetes mellitus and its complications. Part 1: diagnosis and classification of diabetes mellitus. Provisional report of a WHO consultation**. *Diabetic medicine* 1998, **15**(7):539-553.

2. Balkau B: **Comment on the provisional report from the WHO consultation. European Group for the Study of Insulin Resistance (EGIR)**. *Diabet med* 1999, **16**:442-443.

3. Expert Panel on Detection E: **Executive summary of the Third Report of the National Cholesterol Education Program (NCEP) expert panel on detection, evaluation, and treatment of high blood cholesterol in adults (Adult Treatment Panel III)**. *Jama* 2001, **285**(19):2486.

4. Einhorn M, FACP, FACE, Daniel: **American College of Endocrinology position statement on the insulin resistance syndrome**. *Endocrine Practice* 2003, **9**(Supplement 2):5-21.

5. Alberti KGMM, Zimmet P, Shaw J: **Metabolic syndrome—a new world‐wide definition. A consensus statement from the international diabetes federation**. *Diabetic medicine* 2006, **23**(5):469-480.
