# Supplementary material for: Social determinants of common metabolic risk factors (high blood pressure, high blood sugar, high body mass index and high waist-hip ratio) of major non-communicable diseases in South Asia region: a systematic review protocol
Source: Syst Rev. 2017 Sep 7;6:183. doi: 10.1186/s13643-017-0576-6 (PMC5590129; doi:10.1186/s13643-017-0576-6)
Supplement: Supplementary file 2 — Appendix II: PRISMA-P checklist for the systematic review (DOCX 21 kb) [file 13643_2017_576_MOESM2_ESM.docx]

Additional file 2: Appendix II: PRISMA-P checklist for the systematic review

**PRISMA-P (Preferred Reporting Items for Systematic review and Meta-Analysis Protocols) 2015 checklist: recommended items to address in a systematic review protocol***

| Section and topic | Item No | Checklist item | Line number(s) |
| --- | --- | --- | --- |
| ADMINISTRATIVE INFORMATION | | |  |
| Title: |  |  |  |
| Identification | 1a | Social Determinants of common NCD metabolic risk factors (high blood-pressure, high blood sugar and high Body Mass Index) in South Asian Region: a systematic review protocol (SODEN Study) | 6-8 |
| Registration | 2 | The protocol has been registered with PROSPERO (CRD42017067212) | 112 |
| Authors: |  |  |  |
| Contact | 3a | Shiva Raj Mishra, Nepal Development Society, Kathmandu, Nepal, email: shivarajmishra@gmail.com | 33-36 |
| Contributions | 3b | SRS conceived and drafted the initial manuscript. SRM, RP, AM, JF, KW, DL and AV all critically reviewed and revised the initial manuscript. SRS and KW prepared the final manuscript. All authors read and approved the final manuscript. | 211-213 |
| Amendments | 4 | The protocol will be only amended after discussing with majority of the review team members based on the emerging evidences and/or to avoid duplication affecting the objective of the review. |  |
| Support: |  |  |  |
| Sources | 5a | There is no any external funding to report. | 210 |
| Sponsor | 5b | NA |  |
| Role of sponsor or funder | 5c | NA |  |
| INTRODUCTION | | |  |
| Rationale | 6 | There is a need to understand social determinants of common metabolic risk factors of major NCDs for systematic action in the South Asia region using system science approach and tool. However, there is a scarcity of such comprehensive systematic reviews on social determinants of common metabolic risk factors in this region. This systematic review will first identify the common social determinants of major NCDs and then map their causal pathways through which they operate to perpetuate common metabolic risk factors (high blood pressure, high blood sugar, high BMI and high WHR) among adult population in South Asia. | 101-107 |
| Objectives | 7 | The objective of this systematic review is to identify the common social determinants of major NCDs and then map their causal pathways through which they operate to perpetuate common metabolic risk factors (high blood pressure, high blood sugar, high BMI and high WHR) among adult population in South Asia. | 105-107 |
| METHODS | | |  |
| Inclusion criteria | 8 | Inclusion criteria for selection of studies and grey literature will be as follows: a) primary research and grey literature published between 2000 and 2016, b) studies among adult population aged 20-65 from South Asian region (Nepal, India, Bangladesh, Pakistan, Sri Lanka, Bhutan, Maldives, Afghanistan) and c) studies and literature that have generated empirical evidences on proximal and/or distal determinants of the selected NCDs risk factors. Both published and unpublished, grey and peer reviewed, qualitative or quantitative studies will be included. Any study outside of the South-Asia region and not mentioning at least one of the proximal and/or one of the distal determinants from the SDH framework will be excluded | 121-129 |
| Information sources | 9 | Relevant published articles and grey literatures will be searched through PubMed, Web of Science and ProQuest central. | 116-117 |
| Search strategy | 10 | We will search for relevant published articles and grey literature through PubMed, Embase, Web of Science and ProQuest Central using sequence of key words listed in table 1. A detailed search strategy using Boolean operators and contractions for key search terms will be developed in consultation with a librarian at the Massey University library at Wellington. | 116-119 |
| Study records: |  |  |  |
| Data management | 11a | Data will be extracted from the final list of articles by the research team in an illustrative matrix as shown in Table 2. | 151-152 |
| Selection process | 11b | Studies will be primarily screened on the basis of inclusion criteria. Two independent reviewers (SRS and KW) will review the title and abstract checking all the inclusion criteria. We will use Covidence (https://www.covidence.org/), a Cochrane Technology Platform tool to systematize the process. Secondary screening will involve full-text review, review of methods including selected cut-off values for metabolic risk factors [Appendix I] and review of findings and discussion section for causal pathway analysis on social determinants of NCD risk factors. The team will exclude the duplicated articles and then finalize the list for systematic review. | 131-137 |
| Data collection process | 11c | The selected articles will be assessed for their quality using QUALSYST tool. Two independent researchers (SRS and KW) will assess quality of the studies and exclude papers with low quality score (Inter-rater agreement score of <0.55). The final list will be agreed upon by both researchers (SRS and KW) for data extraction and analysis.  Data will be extracted from the final list of articles by the research team in an illustrative matrix as shown in Table 2. | 139  147-149  151-152 |
| Data items | 12 | Social determinants of health (SDH) framework proposed by World Health Organization will be utilized as a guiding framework. In the framework, proximal determinants include behavioral risk factors, psychosocial, material circumstances, community capital and cohesion, health services whereas distal determinants include socio-economic context and social positioning. | 85-86  89-92 |
| Outcomes and prioritization | 13 | The main outcome would be causal loop diagrams of social determinants of common metabolic risk factors that will provide insights on how the social determinants are linked with each other in perpetuating NCD problem for the South Asia region context. The review will have much needed policy and programmatic implications in the current situation of increasing burden of NCD in South Asia. | 195-199 |
| Risk of bias in individual studies | 14 | Risk of bias in individual studies will be minimized through assuring quality of the research paper as stated in the protocol. | 139-149 |
| Data synthesis | 15 | Data will be extracted from the final list of articles by the research team in an illustrative matrix as shown in Table 2.  Thematic analysis will be performed to identify patterns/themes across the extracted data/codes and stream out common pathways from determinants to risk factors of NCD simultaneously to data extraction.  The identified causal linkages/pathways will be further consolidated as per the social determinants of health framework and mapped in the form of Causal Loop Diagram (CLD). | 151-152  155-157  160-161 |
| Limitation of the review | 16 | This review will be limited to articles written in English language and identified through three online databases. | 199-200 |
| Confidence in cumulative evidence | 17 | NA |  |

*** It is strongly recommended that this checklist be read in conjunction with the PRISMA-P Explanation and Elaboration (cite when available) for important clarification on the items. Amendments to a review protocol should be tracked and dated. The copyright for PRISMA-P (including checklist) is held by the PRISMA-P Group and is distributed under a Creative Commons Attribution Licence 4.0.**

*From: Shamseer L, Moher D, Clarke M, Ghersi D, Liberati A, Petticrew M, Shekelle P, Stewart L, PRISMA-P Group. Preferred reporting items for systematic review and meta-analysis protocols (PRISMA-P) 2015: elaboration and explanation. BMJ. 2015 Jan 2;349(jan02 1):g7647.*
