# Supplementary material for: Social determinants of common metabolic risk factors (high blood pressure, high blood sugar, high body mass index and high waist-hip ratio) of major non-communicable diseases in South Asia region: a systematic review protocol
Source: Syst Rev. 2017 Sep 7;6:183. doi: 10.1186/s13643-017-0576-6 (PMC5590129; doi:10.1186/s13643-017-0576-6)
Supplement: Supplementary file 3 — Appendix III: List of proximal and distal determinants (based on SDH framework) (DOCX 16 kb) [file 13643_2017_576_MOESM3_ESM.docx]

**Additional file 3: Appendix III: List of proximal and distal determinants (based on SDH framework)** [1]

***Dependent or Outcome variables***

Metabolic Risk Factors (high blood pressure, high blood sugar, high Body Mass Index and high waist-hip ratio)

***List of Proximal Determinants***

Life style/behavioural related factors

- Smoking habit
- Alcoholism
- Physical exercise
- High salt intake
- Food habit

Community capital/Cohesion

- Network, Trust, Support, Collaboration

Material circumstances

- Communities and neighbourhood environment like violence, poverty level, education level and housing status, parks and recreations, roads, public transport
- Material availability and adequacy for quality of life and wellbeing (housing, water, sanitation, fuel wood, shops)

Psychosocial factors

- Individual Stress especially work and family related
- Social stress relating to living conditions and social cohesion

Health System related factors

Health System Structure

- Health System policy structure for NCD Prevention
- Health System Coverage and Access Issues
- Addressing NCD through overstretched health system
- Governance and corruption
- Inter-department committees for NCD prevention

Funding of NCD primary prevention actions

- Policies and strategies and actions specific to NCD
- Budget allocation per year for NCD prevention
- Donor support/fund for NCD prevention

Multisector collaboration mechanism

Engagement with private sectors (Health, education, development)

- Integrated budgeting and accounting
  Policy and programs for NCD prevention and control in district
- Effective law formulation at local level especially relating to tobacco and alcohol
- Interaction between policy and district health system for NCD response

District/Local Health system functioning

- NCD prevention programs and services including Quality of care
- Skills of Human Resources relating to NCD prevention
- Medicines and Technologies
- Recording and reporting
- Intersectoral collaboration
- Intersectoral actions if any (health, food, occupation, education, social development, law) including District/Municipality/Village planning and budgeting
- Coordination with Municipality and Village Offices for discharging NCD prevention actions

***List of Distal Determinants***

Socio economic factors

- Ethnicity
- Education
- Occupation
- Income/Wealth Index

Socio-political Context

- Political commitment and action
  - Enforcing policy control on harmful products like tobacco and alcohol (including controlling aggressive marketing and political lobbying and increasing tax)
- Political Will for “Health in all” policies
- Gender discrimination
- Social exclusion
- Poverty

Social sector policies and governance

- Policies relating to health, food, occupation, education, social security and urban development affecting NCD prevalence
- Performance of bureaucracy (Social sector)
- Political influence and corruption for effective functioning of social sector
- Inter-ministerial and interdepartmental coordination

References:

1. Solar O, Irwin A: **A conceptual framework for action on the social determinants of health**. In: *Social Determinants of Health Discussion Paper 2 (Policy and Practice).* Geneva: World Health Organization; 2010.
